# Supplementary material for: Hybrid identification for Glycine max and Glycine soja with SSR markers and analysis of salt tolerance
Source: PeerJ. 2019 Feb 19;7:e6483. doi: 10.7717/peerj.6483 (PMC6385681; doi:10.7717/peerj.6483)
Supplement: Supplemental Information 1 — Primer sequences were randomly selected from soybean public databases (http://soybase.org/resources/ssr.php). Among them, 24 SSR primers (shown in boldface) were used to verify the stability of the obtained system (the design No.7 in Table S2), 18 SSR primers (shown in italics) were used to screen polymorphic primers between parents of different cross combinations. [file peerj-07-6483-s001.doc]

**Table S1** Primers used in this work

| **Primer names** | **Sequences** |
| --- | --- |
| **Satt519** | **F:** GGATTTCAAAGAATGAACACAGA |
|  | **R:** CCGCAAGGTTACGAACTGCTCGAA |
| **Satt467** | **F:** GCGAAGAGCTACATCTAACACAATTCAA |
|  | **R:** GCGTTAAGCACGGTCATATTTTCTCATA |
| **Satt474** | **F:** GCGAAATTTGGAAATGACATCTTAGAA |
|  | **R:** GCGACGGGAGAAATTGGATGTGAAGAA |
| **Sat_367** | **F:** GCGGATATGCCACTTCTCTCGTGAC |
|  | **R:** GCGGAATAGTTGCCAAACAATAATC |
| **Sat_311** | **F:** GGGGGAACCACAAAAATCTTAATC |
|  | **R:** GTTGAAGCTCAGGCTGTGATGAAT |
| **Satt432** | **F:** GCCAGGTTGTGTTCTTGAGATA |
|  | **R:** TTCACCGAATATTCTTTTAGGTC |
| **Satt444** | **F:** TGCAAAAATACGGGTTCATAAT |
|  | **R:** AGAGGAAGCGAGACTAATAGAAG |
| **Satt168** | **F:** CGCTTGCCCAAAAATTAATAGTA |
|  | **R:** CCATTCTCCAACCTCAATCTTATAT |
| **Satt726** | **F:** GCGTTTTTAGTATGGATAATGTTTT |
|  | **R:** GCGAAGGGACAAGAGTGAT |
| **Satt161** | **F:** GGGTATATCAACATATCTTCACCTTTTT |
|  | **R:** GGGCTGCTTGTTAATGTTTTGTAGA |
| **Satt682** | **F:**GCGTTTAAACTATTTTGTAATTTATTGTGAA |
|  | **R:**GCGGGGGAAATATTAGAAAAGTGATACAT |
| **Satt_153** | **F:** GGGTTATATCAGTTTTTCTTTTTGTT |
|  | **R:** CCATCCTCGTTAGCATCTAT |
| **Satt_264** | **F:** CCTTTTGACAATTATGGCATATA |
|  | **R:** GCATAGAAGGGCATCATTCAGAT |
| **Satt556** | **F:** GCGATAAAACCCGATAAATAA |
|  | **R:** GCGTTGTGCACCTTGTTTTCT |
| **Satt194** | **F:** GGGCCCAACTGATATTTAATTGTAA |
|  | **R:** GCGCTTTGTGTTCCGATTTTGAT |
| **Satt_207** | **F:** GCGTTTTTCTCATTTTGATTCCTAAAC |
|  | **R:** GCGATTGTGATTGTAGTCCCTAAA |
| **Satt286** | **F:** GCGGCGTTAATTTATGCCGGAAA |
|  | **R:** GCGTTTGGTCTAGAATAGTTCTCA |
| **Sat_332** | **F:** GCGCATCCAGGGCTTGCAACAAAG |
|  | **R:** GCGGTCCTTATATATGGAAGATCA |
| **Satt254** | **F:** GGGCATGCATAATCTCTCTGCAAC |
|  | **R:** GCGGGGATATTTATTGATTTAGT |
| **Satt147** | **F:** CCATCCCTTCCTCCAAATAGAT |
|  | **R:** CTTCCACACCCTAGTTTAGTGACAA |
| **Satt447** | **F:** CGAAACTACGGTTGATTAT |
|  | **R:** TCCAAACACTGTCCTTCTT |
| **Satt_220** | **F:** GAGGAGGATCCCAAGGTAATAAT |
|  | **R:** GCGCATGGAGAAAAGAAGAG |
| **Satt708** | **F:** GCGCAATTTTAAGAGATTTTCGGGATAA |
|  | **R:** GCGACTCGGTTGATTTTTTTTTCAATTTTTT |
| **Satt368** | **F:** GCGAGTGCAAGAAAAAGGGAGAAAATA |
|  | **R:** GCGCTTTTCTTTCCTTTTTTTTCTCTT |
| *Satt682* | **F:**GCGTTTAAACTATTTTGTAATTTATTGTGAA |
|  | **R:**GCGGGGGAAATATTAGAAAAGTGATACAT |
| *Satt70* | **F:** TGGGATCCCTTATCCATGTG |
|  | **R:** ACACCTCTCATTCACGGGAC |
| *Satt368* | **F:** GCGAGTGCAAGAAAAAGGGAGAAAATA |
|  | **R:** GCGCTTTTCTTTCCTTTTTTTTCTCTT |
| *Satt440* | **F:** TGAGAACGTTTGAAAAGAGAT |
|  | **R:** GAAGAGATTAAGCATAAAGAATACTT |
| *Sat_246* | **F:**GCGCATGGTTTACAGATTACTTTATTTTCTA |
|  | **R:**GCGGCAATCATTTAAATTTATAATGATATA |
| *Sat_240* | **F:** GCGCCCTTTGGATTTTATTGC |
|  | **R:** TTTCACCTTCCCTTCTTTTGA |
| *Sat_262* | **F:** GCGCCCCATTAATGTTAACACA |
|  | **R:** GCGGAGTTCAACGCATTCACCTT |
| *Satt649* | **F:** TTACTGGCCGTGTTTACCCGTGTAA |
|  | **R:** GCGGACGTTATAAGATTTTTTTATCATG |
| *Satt170* | **F:** GGGAAATCTAAATAAAATGATGGATAT |
|  | **R:** GGGGTAGTTAAAATTCATCCTTAAAA |
| *Satt242* | **F:** GCGTTGATCAGGTCGATTTTTATTTGT |
|  | **R:** GCGAGTGCCAACTAACTACTTTTATGA |
| *Satt152* | **F:** GCGCTATTCCTATCACAACACA |
|  | **R:** TAGGGTTGTCACTGTTTTGTTCTTA |
| *Satt102* | **F:** GATTTCCAACTTATGAATCTTAATAA |
|  | **R:** GAAAATACATAAGAGCATAATGTCA |
| *Sat_359* | **F:** GCGAGAAAATAATCCTGCTCAAG |
|  | **R:** GCGTTTAAGTCCAATAACAAAGATAAC |
| *Sat_276* | **F:** GCGGACGGTAAGGACTATTTATGATA |
|  | **R:** GCGTCAGATGAAAAAAAATAAGATAC |
| *Sat_393* | **F:** CAAGCCCATAAACGAAATAA |
|  | **R:** GCTCGGCTTGGCTTGTTTACTA |
| *Satt530* | **F:** TGGACGTGAATAGGCAGTTC |
|  | **R:** TTGTTTTTCCTTTTGATATCATGC |
| *Satt348* | **F:** GCGCTTAGTAATGGTTCCCACAGATAA |
|  | **R:** GCGGTGATATCTAGCAACACAA |
| *Satt072* | **F:** GGAAAGAATCAGCAAAAT |
|  | **R:** CCCCCACATAAATAATAAA |
