# Supplementary material for: Hybrid identification for Glycine max and Glycine soja with SSR markers and analysis of salt tolerance
Source: PeerJ. 2019 Feb 19;7:e6483. doi: 10.7717/peerj.6483 (PMC6385681; doi:10.7717/peerj.6483)
Supplement: Supplemental Information 2 — The orthogonal test was performed at 5 factors (Mg2+ concentration, primer concentration, dNTPs concentration, Taq enzyme concentration and DNA template concentration,) and 4 levels for establishing the more appropriate SSR-PCR reaction system. [file peerj-07-6483-s002.doc]

**Table S2 The orthogonal experimental design (L_16_(4^5^)) for construction of soybean SSR-PCR reaction system**

| No. | Factors | | | | |
| --- | --- | --- | --- | --- | --- |
|  | Mg^2+^ (mmol/L) | Primer (μmol/L ) | dNTPs (μmol/L ) | Taq enzyme（U） | DNA Template (ng/10 μl) |
| 1 | 1.5 | 0.5 | 120 | 0.5 | 10 |
| 2 | 1.5 | 1.0 | 160 | 0.6 | 20 |
| 3 | 1.5 | 1.5 | 200 | 0.7 | 30 |
| 4 | 1.5 | 2.0 | 240 | 0.8 | 40 |
| 5 | 2.0 | 0.5 | 160 | 0.7 | 40 |
| 6 | 2.0 | 1.0 | 120 | 0.8 | 20 |
| 7 | 2.0 | 1.5 | 240 | 0.5 | 30 |
| 8 | 2.0 | 2.0 | 200 | 0.6 | 10 |
| 9 | 2.5 | 0.5 | 200 | 0.8 | 40 |
| 10 | 2.5 | 1.0 | 240 | 0.7 | 30 |
| 11 | 2.5 | 1.5 | 120 | 0.6 | 20 |
| 12 | 2.5 | 2.0 | 160 | 0.5 | 10 |
| 13 | 3.0 | 0.5 | 240 | 0.6 | 40 |
| 14 | 3.0 | 1.0 | 200 | 0.5 | 30 |
| 15 | 3.0 | 1.5 | 160 | 0.8 | 20 |
| 16 | 3.0 | 2.0 | 120 | 0.7 | 10 |
