# Supplementary material for: Hybrid identification for Glycine max and Glycine soja with SSR markers and analysis of salt tolerance
Source: PeerJ. 2019 Feb 19;7:e6483. doi: 10.7717/peerj.6483 (PMC6385681; doi:10.7717/peerj.6483)
Supplement: Supplemental Information 3 — The numbers of parental polymorphic SSR primers were counted according to the banding pattern of male and female parents in Figure S2 in different soybean cross combinations (A–K). [file peerj-07-6483-s003.doc]

**Table S3 Selection of the co-dominant polymorphic primers among the soybean parents of different cross combinations**

| Soybean cross combinations | No. of SSR markers | No. of polymorphic primers |
| --- | --- | --- |
| A | 18 | 11 |
| B | 18 | 9 |
| C | 18 | 6 |
| D | 18 | 9 |
| E | 18 | 11 |
| F | 18 | 7 |
| G | 15 | 5 |
| H | 18 | 7 |
| I | 18 | 5 |
| J | 18 | 8 |
| K | 18 | 6 |
