# Supplementary figures and images for: Hybrid identification for Glycine max and Glycine soja with SSR markers and analysis of salt tolerance
Source: PeerJ. 2019 Feb 19;7:e6483. doi: 10.7717/peerj.6483 (PMC6385681; doi:10.7717/peerj.6483)

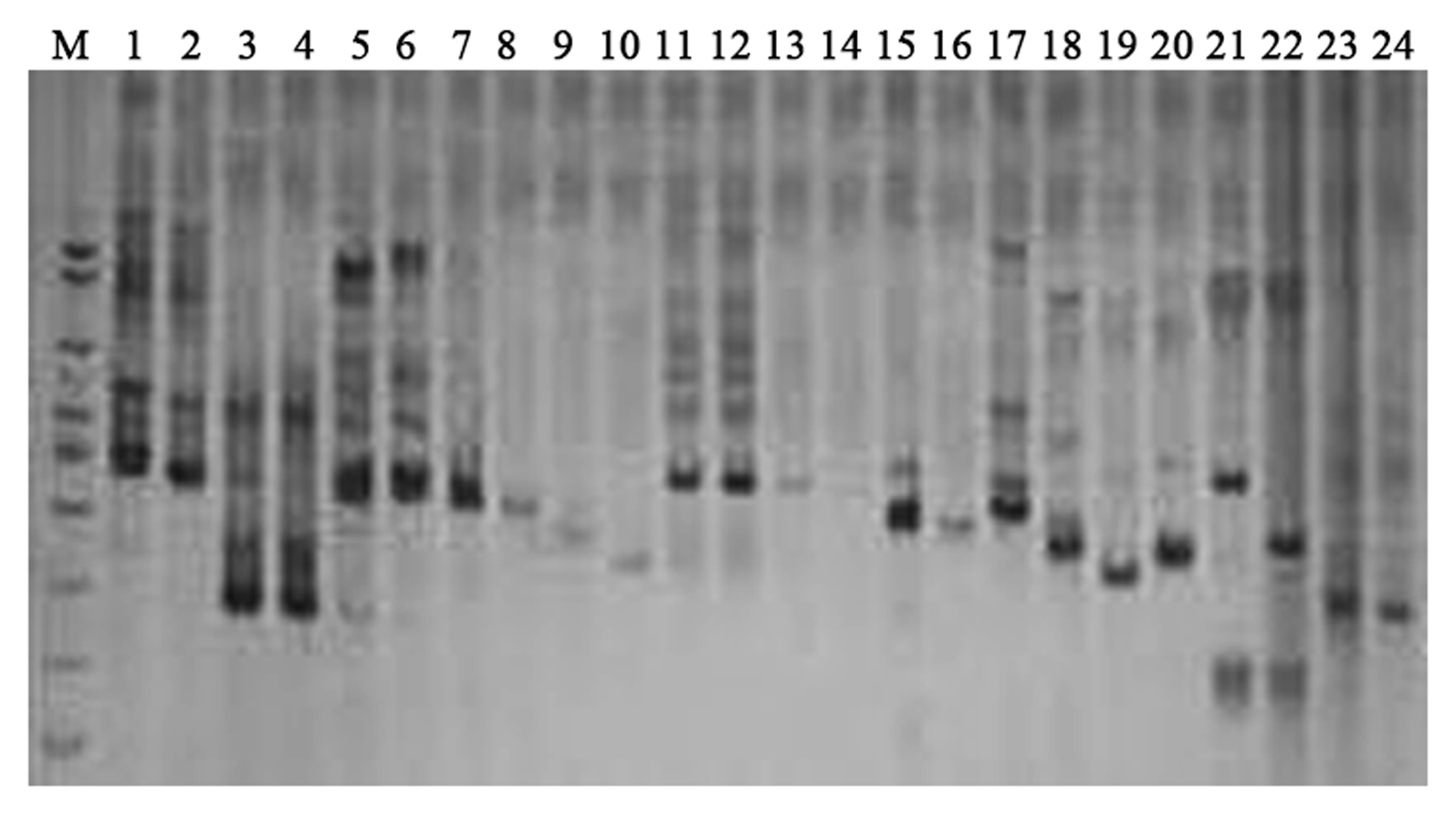

Supplement: Supplemental Information 4 — M: DNA marker; Lane 1–24 represented SSR primers Satt519, Satt467, Satt474, Sat-367, Sat-311, Satt432, Satt444, Satt168, Satt726, Satt161, Satt682, Satt-153, Satt-264, Satt556, Satt194, Satt-207, Satt286, Sat-332 , Satt254, Satt147, Satt447, Satt-220, Satt708 and Satt368, respectively. [file peerj-07-6483-s004.png]

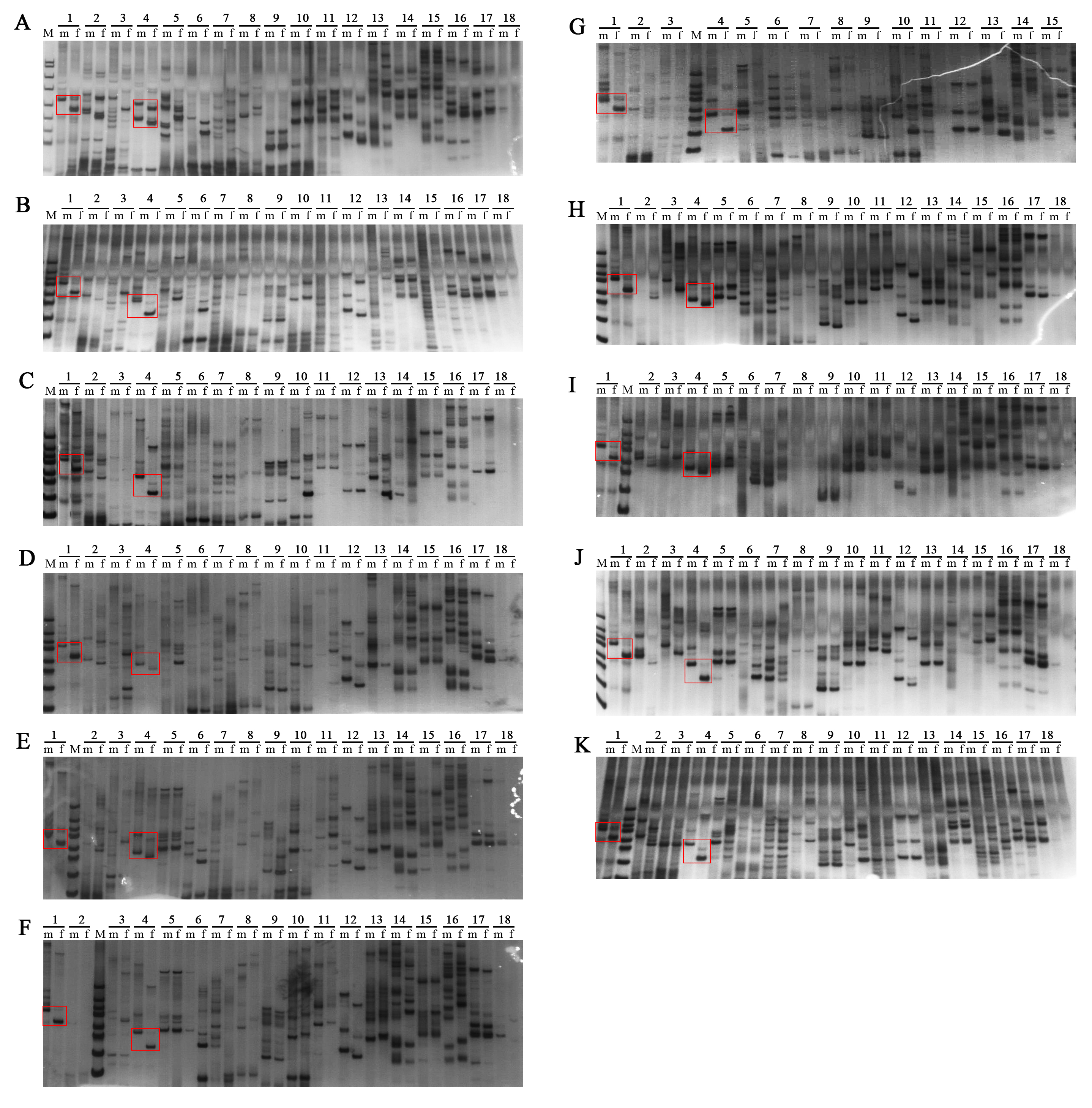

Supplement: Supplemental Information 5 — A–K: The 11 soybean cross combinations (corresponding to Table 1); M: DNA marker; Lane 1–18 representing SSR primer Satt682, Satt70, Satt368, Satt440, Sat-246, Sat-240, Sat-262, Satt649, Satt170, Satt242, Satt152, Satt102, Sat-359, Sat-276, Sat-393, Satt530, Satt348 and Satt072, respectively; m: male parent; f: female parent. Satt682 and Satt440 displayed parental co-dominant polymorphism among all the parental lines in different combinations, and the polymorphic bands have been marked with red boxes. [file peerj-07-6483-s005.png]
